# Bis[(4-methyl­phen­yl)ethyn­yl] telluride

**DOI:** 10.1107/S1600536810006264

**Published:** 2010-02-24

**Authors:** Ignez Caracelli, Julio Zukerman-Schpector, Jesus M. Pena, Hélio A. Stefani, Edward R. T. Tiekink

**Affiliations:** a BioMat-Physics Department, Univ Estadual Paulista, UNESP, 17033-360 Bauru, SP, Brazil; b Department of Chemistry, Universidade Federal de São Carlos, 13565-905 São Carlos, SP, Brazil; c Departamento de Farmácia, Faculdade de Ciências Farmacêuticas, Universidade de São Paulo, São Paulo-SP, Brazil; d Department of Chemistry, University of Malaya, Kuala Lumpur 50603, Malaysia

## Abstract

The tellurium atom in the title bis-ethynyl telluride, Te(C_9_H_7_)_2_ or C_18_H_14_Te, is located on a crystallographic twofold axis, the C—Te—C angle being 92.23 (15)°. The dihedral angle between the rings is 87.27 (7)°. In the crystal structure, mol­ecules are connected in chains parallel to the *b* axis and mediated by C—H⋯π inter­actions.

## Related literature

For the synthesis of bis-ethynyl tellurides, see: Gedridge *et al.* (1992 ▶); Engman & Stern (1993 ▶). For background to the motivation of studies into tellurium chemistry, see: Petragnani & Stefani (2007 ▶); Zukerman-Schpector *et al.* (2008 ▶). For related structures, see: Jones & Ruthe (2006 ▶). For searching the Cambridge Structural Database, see: Bruno *et al.* (2002 ▶). For background to Te⋯π inter­actions, see: Tiekink & Zukerman-Schpector (2009 ▶); Zukerman-Schpector & Haiduc (2002 ▶).
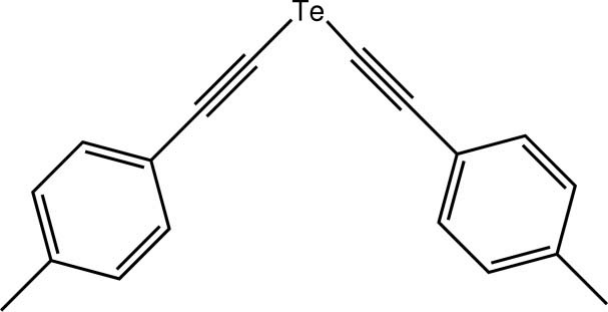


## Experimental

### 

#### Crystal data

C_18_H_14_Te
*M* *_r_* = 357.89
Monoclinic, 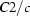

*a* = 25.8462 (8) Å
*b* = 4.8902 (2) Å
*c* = 11.3764 (3) Å
β = 100.316 (2)°
*V* = 1414.65 (8) Å^3^
*Z* = 4
Mo *K*α radiation
μ = 2.09 mm^−1^
*T* = 100 K
0.27 × 0.13 × 0.09 mm

#### Data collection

Bruker SMART APEXII diffractometer
Absorption correction: multi-scan (*SADABS*; Sheldrick, 1996 ▶) *T*_min_ = 0.617, *T*_max_ = 0.746
5433 measured reflections
1443 independent reflections
1350 reflections with *I* > 2σ(*I*)
*R*_int_ = 0.020

#### Refinement

*R*[*F* ^2^ > 2σ(*F* ^2^)] = 0.019
*wR*(*F* ^2^) = 0.055
*S* = 1.20
1443 reflections
88 parameters
H-atom parameters constrained
Δρ_max_ = 0.74 e Å^−3^
Δρ_min_ = −0.72 e Å^−3^

### 

Data collection: *APEX2* (Bruker, 2007 ▶); cell refinement: *SAINT* (Bruker, 2007 ▶); data reduction: *SAINT*; program(s) used to solve structure: *SIR97* (Altomare *et al.*, 1999 ▶); program(s) used to refine structure: *SHELXL97* (Sheldrick, 2008 ▶); molecular graphics: *ORTEP-3* (Farrugia, 1997 ▶) and *DIAMOND* (Brandenburg, 2006 ▶); software used to prepare material for publication: *WinGX* (Farrugia, 1999 ▶).

## Supplementary Material

Crystal structure: contains datablocks global, I. DOI: 10.1107/S1600536810006264/hg2646sup1.cif

Structure factors: contains datablocks I. DOI: 10.1107/S1600536810006264/hg2646Isup2.hkl

Additional supplementary materials:  crystallographic information; 3D view; checkCIF report

## Figures and Tables

**Table 1:** Hydrogen-bond geometry (Å, °) *Cg* is the centroid of the C3–C8 ring.

*D*—H⋯*A*	*D*—H	H⋯*A*	*D*⋯*A*	*D*—H⋯*A*
C9—H9a⋯*Cg*^i^	0.98	2.62	3.573 (3)	163

Symmetry code: (i) 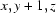.

**Fig. 1.**
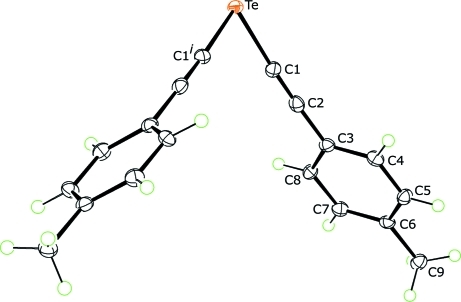
The molecular structure of (I) showing atom labelling scheme and displacement ellipsoids at the 50% probability level (arbitrary spheres for the H atoms). Symmetry operation i: -x, y, 3/2-z.

**Fig. 2.**
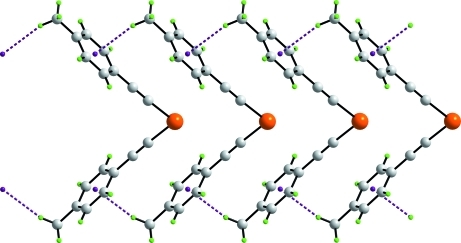
Supramolecular chain aligned along the b axis in (I) sustained by C–H···π interactions shown as orange dashed lines. Colour code: Te, purple; C, grey; and H, green.

## supplementary crystallographic information

### Comment

Carbon–carbon bond formation for the preparation of symmetrical and unsymmetrical 1,3-diyne compounds is one of the most useful and important tools in modern organic chemistry. The construction of 1,3-diynes can be achieved either by intermolecular or intramolecular coupling of two similar or dissimilar alkynylic functionalities in the presence of organometallic complexes. However, the synthesis and use of bis-ethynyl tellurides are scarcely described in the literature (Gedridge *et al.*, 1992, Engman & Stern, 1993) and their use in the detelluration reaction to afford 1,3-diynes is unknown until now. As part of our ongoing research into tellurium chemistry (Petragnani & Stefani, 2007; Zukerman-Schpector *et al.*, 2008), the title compound, (I), was synthesized and its crystal structure determined.

The C—Te—C in (I), Fig. 1, angle of 92.23 (15) ° is close to the smallest value found for related diorganotellurium compounds, i.e. 92.30 (14) ° for Te[C(H)═C(H)Ph]_2_ (Jones & Ruthe, 2006). A search in the CSD (Bruno *et al.* 2002) showed 225 hits for related compounds and a mean value of 96.0 ° for the C—Te(II)—C angle.

The molecules are linked in chains parallel to the *b* axis mediated in a large part through C–H···π interactions, Table 1 and Fig. 1. Short intermolecular Te–C interactions [*e*.*g*. Te···C2^ii^ = 3.541 (3) Å for *ii*: *x*, -1+ *y*, *z*], indicative of Te···π interactions (Zukerman-Schpector & Haiduc, 2002; Tiekink & Zukerman-Schpector, 2009), are also noted as contributing to the stability of the chain.

### Experimental

To a stirred solution of 1-ethynyl-4-methylbenzene (0.35 g, 3.0 mmol) in THF (10 ml), *n*-BuLi (1.2 ml, 2.5 M, 3.0 mmol) was added dropwise at 195 K. After 20 min., freshly crushed tellurium powder (0.38 g, 3.0 mmol) was added in one lot while a stream of argon was passed through the open flask. The cooling bath was then removed to bring the reaction medium to room temperature. When almost all the tellurium was consumed, the reaction mixture was again cooled to 195 K. Then a solution of bromine (0.48 g, 3.0 mmol) in dry benzene (5 ml) was added dropwise, and stirring was continued for 15 min. The reaction mixture was hydrolyzed at 195 K by addition of water (5 ml). Dilution with water (20 ml) at room temperature, extraction with dichloromethane (2 x 15 ml), drying (MgSO_4_), and flash chromatography (1/4 dichloromethane/hexane) afforded 0.90 g (62% yield) of the title compound as yellow crystals, m.pt. 400–401 K.

### Refinement

The H atoms were geometrically placed (C–H = 0.95–0.98 Å) and refined as riding with *U_iso_*(H) = 1.2-1.5*U_eq_*(C).

### Crystal data

**Table:**

C_18_H_14_Te	*F*(000) = 696
*M**_r_* = 357.89	*D*_x_ = 1.680 Mg m^−^^3^
Monoclinic, *C*2/*c*	Mo *K*α radiation, λ = 0.71073 Å
Hall symbol: -C 2yc	Cell parameters from 4746 reflections
*a* = 25.8462 (8) Å	θ = 2.2–27.7°
*b* = 4.8902 (2) Å	µ = 2.09 mm^−^^1^
*c* = 11.3764 (3) Å	*T* = 100 K
β = 100.316 (2)°	Block, pale-yellow
*V* = 1414.65 (8) Å^3^	0.27 × 0.13 × 0.09 mm
*Z* = 4	

### Data collection

**Table:**

Bruker SMART APEXII diffractometer	1443 independent reflections
Radiation source: sealed tube	1350 reflections with *I* > 2σ(*I*)
graphite	*R*_int_ = 0.020
φ and ω scans	θ_max_ = 26.5°, θ_min_ = 1.6°
Absorption correction: multi-scan (*SADABS*; Sheldrick, 1996)	*h* = −32→32
*T*_min_ = 0.617, *T*_max_ = 0.746	*k* = −6→5
5433 measured reflections	*l* = −14→14

### Refinement

**Table:**

Refinement on *F*^2^	Primary atom site location: structure-invariant direct methods
Least-squares matrix: full	Secondary atom site location: difference Fourier map
*R*[*F*^2^ > 2σ(*F*^2^)] = 0.019	Hydrogen site location: inferred from neighbouring sites
*wR*(*F*^2^) = 0.055	H-atom parameters constrained
*S* = 1.20	*w* = 1/[σ^2^(*F*_o_^2^) + (0.019*P*)^2^ + 5.1394*P*] where *P* = (*F*_o_^2^ + 2*F*_c_^2^)/3
1443 reflections	(Δ/σ)_max_ < 0.001
88 parameters	Δρ_max_ = 0.74 e Å^−^^3^
0 restraints	Δρ_min_ = −0.72 e Å^−^^3^

### Special details

**Table:**

Geometry. All s.u.'s (except the s.u. in the dihedral angle between two l.s. planes) are estimated using the full covariance matrix. The cell s.u.'s are taken into account individually in the estimation of s.u.'s in distances, angles and torsion angles; correlations between s.u.'s in cell parameters are only used when they are defined by crystal symmetry. An approximate (isotropic) treatment of cell s.u.'s is used for estimating s.u.'s involving l.s. planes.
Refinement. Refinement of *F*^2^ against ALL reflections. The weighted *R*-factor wR and goodness of fit *S* are based on *F*^2^, conventional *R*-factors *R* are based on *F*, with *F* set to zero for negative *F*^2^. The threshold expression of *F*^2^ > σ(*F*^2^) is used only for calculating *R*-factors(gt) etc. and is not relevant to the choice of reflections for refinement. *R*-factors based on *F*^2^ are statistically about twice as large as those based on *F*, and *R*- factors based on ALL data will be even larger.

### Fractional atomic coordinates and isotropic or equivalent isotropic displacement parameters (Å^2^)

**Table:**

	*x*	*y*	*z*	*U*_iso_*/*U*_eq_	
Te	0.0000	0.37974 (5)	0.7500	0.01504 (9)	
C1	0.04373 (10)	0.6695 (6)	0.8528 (2)	0.0161 (5)	
C2	0.07098 (10)	0.8358 (6)	0.9095 (2)	0.0172 (6)	
C3	0.10551 (10)	1.0424 (6)	0.9693 (2)	0.0155 (5)	
C4	0.14665 (10)	1.1424 (6)	0.9162 (2)	0.0173 (6)	
H4	0.1510	1.0759	0.8401	0.021*	
C5	0.18087 (10)	1.3374 (6)	0.9737 (2)	0.0172 (6)	
H5	0.2086	1.4024	0.9365	0.021*	
C6	0.17559 (10)	1.4406 (6)	1.0852 (2)	0.0154 (6)	
C7	0.13423 (10)	1.3430 (6)	1.1371 (2)	0.0179 (6)	
H7	0.1298	1.4119	1.2127	0.022*	
C8	0.09947 (10)	1.1478 (6)	1.0810 (2)	0.0170 (5)	
H8	0.0715	1.0851	1.1181	0.020*	
C9	0.21318 (10)	1.6531 (6)	1.1461 (2)	0.0189 (6)	
H9A	0.1990	1.8358	1.1248	0.028*	
H9B	0.2472	1.6332	1.1202	0.028*	
H9C	0.2179	1.6286	1.2329	0.028*	

### Atomic displacement parameters (Å^2^)

**Table:**

	*U*^11^	*U*^22^	*U*^33^	*U*^12^	*U*^13^	*U*^23^
Te	0.01487 (13)	0.01124 (14)	0.01834 (14)	0.000	0.00119 (9)	0.000
C1	0.0156 (12)	0.0148 (14)	0.0177 (12)	0.0001 (11)	0.0023 (10)	0.0042 (11)
C2	0.0154 (12)	0.0175 (15)	0.0183 (12)	0.0040 (11)	0.0021 (10)	0.0045 (12)
C3	0.0151 (12)	0.0123 (14)	0.0175 (12)	0.0018 (10)	−0.0013 (10)	0.0008 (11)
C4	0.0199 (12)	0.0172 (15)	0.0152 (12)	0.0024 (11)	0.0038 (10)	−0.0026 (12)
C5	0.0179 (12)	0.0163 (15)	0.0179 (13)	−0.0021 (11)	0.0049 (10)	0.0027 (12)
C6	0.0165 (12)	0.0116 (14)	0.0172 (12)	0.0021 (10)	0.0002 (10)	0.0009 (11)
C7	0.0204 (13)	0.0168 (15)	0.0168 (12)	0.0004 (11)	0.0037 (10)	−0.0020 (12)
C8	0.0180 (12)	0.0150 (14)	0.0192 (13)	−0.0016 (11)	0.0069 (10)	0.0037 (12)
C9	0.0182 (12)	0.0177 (15)	0.0199 (13)	−0.0015 (11)	0.0006 (10)	0.0035 (12)

### Geometric parameters (Å, °)

**Table:**

Te—C1	2.044 (3)	C6—C7	1.395 (4)
C1—C2	1.188 (4)	C6—C9	1.504 (4)
C2—C3	1.437 (4)	C7—C8	1.386 (4)
C3—C4	1.402 (4)	C7—H7	0.9500
C3—C8	1.406 (4)	C8—H8	0.9500
C4—C5	1.383 (4)	C9—H9A	0.9800
C4—H4	0.9500	C9—H9B	0.9800
C5—C6	1.394 (4)	C9—H9C	0.9800
C5—H5	0.9500		
			
C1—Te—C1^i^	92.23 (15)	C7—C6—C9	121.5 (2)
C2—C1—Te	176.9 (2)	C8—C7—C6	121.6 (3)
C1—C2—C3	175.3 (3)	C8—C7—H7	119.2
C4—C3—C8	118.5 (3)	C6—C7—H7	119.2
C4—C3—C2	119.7 (3)	C7—C8—C3	120.1 (3)
C8—C3—C2	121.8 (3)	C7—C8—H8	120.0
C5—C4—C3	120.4 (3)	C3—C8—H8	120.0
C5—C4—H4	119.8	C6—C9—H9A	109.5
C3—C4—H4	119.8	C6—C9—H9B	109.5
C4—C5—C6	121.5 (3)	H9A—C9—H9B	109.5
C4—C5—H5	119.3	C6—C9—H9C	109.5
C6—C5—H5	119.3	H9A—C9—H9C	109.5
C5—C6—C7	117.9 (3)	H9B—C9—H9C	109.5
C5—C6—C9	120.6 (2)		
			
C8—C3—C4—C5	1.0 (4)	C5—C6—C7—C8	0.5 (4)
C2—C3—C4—C5	−178.7 (3)	C9—C6—C7—C8	179.8 (3)
C3—C4—C5—C6	−0.2 (4)	C6—C7—C8—C3	0.2 (4)
C4—C5—C6—C7	−0.6 (4)	C4—C3—C8—C7	−1.0 (4)
C4—C5—C6—C9	−179.9 (3)	C2—C3—C8—C7	178.7 (3)

Symmetry codes: (i) −*x*, *y*, −*z*+3/2.

### Hydrogen-bond geometry (Å, °)

**Table:**

Cg is the centroid of the C3–C8 ring.

**Table:**

*D*—H···*A*	*D*—H	H···*A*	*D*···*A*	*D*—H···*A*
C9—H9a···Cg^ii^	0.98	2.62	3.573 (3)	163

Symmetry codes: (ii) *x*, *y*+1, *z*.
